# Case series: Superficial lipomatous hamartoma in dogs and cats is a previously unreported NLCS-like dermal lesion

**DOI:** 10.3389/fvets.2026.1891605

**Published:** 2026-10-07

**Authors:** Laura Kästner, Julia Marchewski, Manfred Henrich

**Affiliations:** Faculty of Veterinary Medicine, Institute of Veterinary-Pathology, Justus Liebig University Giessen, Giessen, Germany

**Keywords:** case report, cutaneous hamartoma, nevus lipomatosus cutaneous superficialis, solitary skin lesion, veterinary dermatopathology

## Abstract

In humans nevus lipomatosus cutaneous superficialis (NLCS) is a rare, benign dermal hamartoma characterized by aggregates of adipose tissue within the dermis without any connection to the subcutaneous tissue. Although NLCS is a well-recognized entity, it is considered a rare lesion with limited reports in the human literature. To date, no comparable cases have been reported in dogs and cats. Over the course of one and a half years, six cases (five in dogs, one in a cat), resembling NLCS-like lesions, were examined. In this report, we compare these cases to the human condition regarding macroscopic features, histopathology, sex, and age of the affected patients. In all cases, the dermal lesions were composed of aggregates of well-differentiated adipocytes within the dermis. Given the morphological similarity to the lesion in humans, the term “nevus lipomatosus superficialis-like lesion” might be appropriate. However, given the controversial use of the term ‘nevus’ in veterinary medicine and a potential for confusion with pigmented lesion, we propose the term “superficial lipomatous hamartoma” for this entity.

## 1 Introduction

In human medicine, nevus lipomatosus cutaneous superficialis (NLCS) is a benign cutaneous hamartoma, histologically characterized by aggregates of mature ectopic adipocytes within the dermal collagen layers (1). It was first described in 1921 by Hoffmann and Zurhelle (2) and is a very rare dermatological condition. In human pathology, the lesion can be classified into two clinical subtypes: a classical type consisting of multiple grouped nodules (also known as Hoffmann-Zurhelle) and a solitary dome-shaped variant (3). This cutaneous malformation is usually not associated with any other symptoms. However, ulceration, pain, and foul odor occasionally occur (3). Grossly, NLCS cannot be distinguished from other cutaneous lesions, making histopathological examination essential for diagnosis. After surgical removal, recurrence is uncommon (4). In veterinary medicine, the term ‘nevus’ is controversial. To avoid confusion with pigmented lesions frequently seen in humans, the WHO International Histological Classification of Tumors of Domestic Animals recommends the term ‘hamartoma’ (5). A hamartoma is defined as a localized accumulation of well-differentiated cells, often arranged irregularly within a specific region, and can be categorized into several subtypes, including epidermal, follicular, sebaceous, apocrine, and fibroadnexal (5). While various cutaneous hamartomas and lipomatous lesions (e.g., dermal lipomas), have been documented in domestic animals, the specific presentation of dermal adipocytes arranged in a pattern resembling the human NLCS has not been clearly described in veterinary medicine.

This case series describes NLCS-like lesions in dogs and a cat, with specific consideration given to the clinical presentation, histopathology, and the age and sex of the affected animals.

## 2 Case reports

In 2025 and 2026, six tissue samples exhibiting features of NLCS were identified among the specimens submitted to the biopsy service of the Institute of Veterinary Pathology at Justus Liebig University Giessen, Germany (Table 1). Each sample underwent a gross examination (Table 1), was processed for histologic examination, and then cut and stained with hematoxylin and eosin (HE) following standard protocols.

**Table 1:** Submitted biopsy specimens.

Case	Species/breed	Sex	Age	Anatomic location	Gross appearance	Histology key findings
1	Dog Labrador retriever	Female	5 years	Axilla	1 cm × 1 cm × 1.5 cm, cut surface: firm and white	Dermal well-differentiated adipocytes, disconnected from the subcutaneous fat, with mild hyperkeratosis
2	Cat European short hair	Male	8 years	Abdomen	1 cm × 1 cm × 0.5 cm, pedunculate, cut surface: white and firm	Dermal well-differentiated adipocytes, disconnected from the subcutaneous fat, mild hyperplastic sebaceous glands
3	Dog Bernese Mountain Dog	Female	3.5 years	Neck	4 cm × 2 cm × 3 cm with 1 cm × 1 cm × 1 cm peduncule, cut surface: beige and elastic	Ulceration with necrosuppurative inflammation (Figure 1), dermal well-differentiated adipocytes, disconnected from the subcutaneous fat
4	Dog Dalmatian	Female	7.5 years	Thigh	2 cm × 1.5 cm × 2 cm, multifocally ulcerated, cut surface: grey and firm	Ulceration with necrosuppurative inflammation, dermal well-differentiated adipocytes, disconnected from the subcutaneous fat
5	Dog French bulldog	Female, spayed	9.6 years	Ear	2 cm × 1 cm × 1 cm, pedunculated, cut surface: white and firm	Necrosuppurative inflammation without Ulceration, dermal well-differentiated adipocytes, disconnected from the subcutaneous fat
6	Dog Mixed breed dog	Female, spayed	9.4 years	Lip	1.5 cm × 0.2 cm × 0.3 cm, cut surface: white to grey and firm	Multinodular, Erosion and Ulceration of the epithelium, lymphoplasmacellular dermatitis, dermal well-differentiated adipocytes, disconnected from the subcutaneous fat (Figure 2)

**Figure 1:**
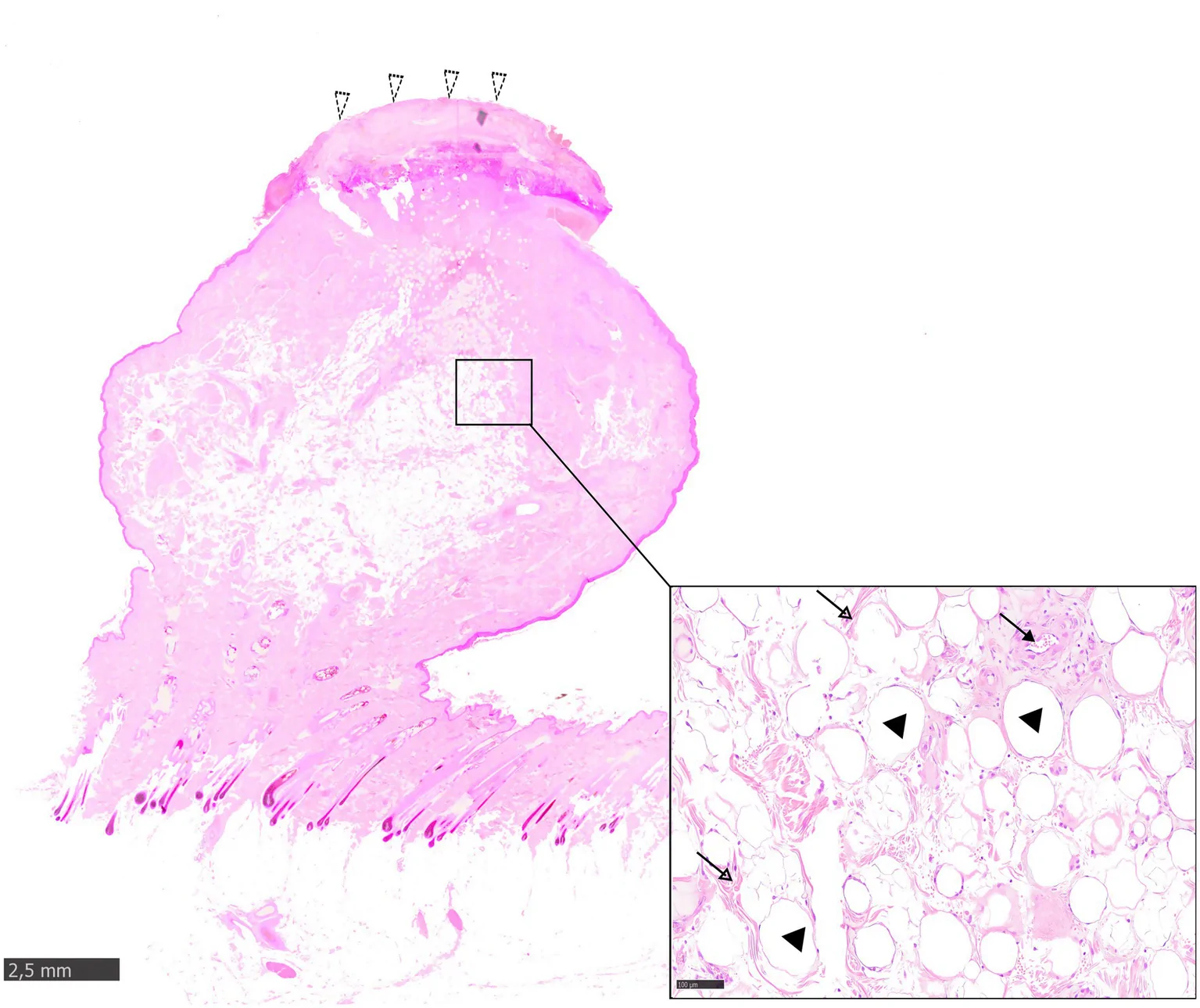
A pedunculated mass consisting of well-differentiated adipocytes separated by collagen fibers. The epithelium is ulcerated with a serocellular crust (empty arrow heads) and necrosuppurative inflammation. (hematoxylin and eosin stain, scale bar: 2.5 mm), Insert: Higher magnification showing well differentiated adipocytes (black arrow heads) adjacent to the epidermis with loosely packed collagen fibers (empty arrows) and small vessels (black arrows). (hematoxylin and eosin stain, scale bar: 250 μm).

**Figure 2:**
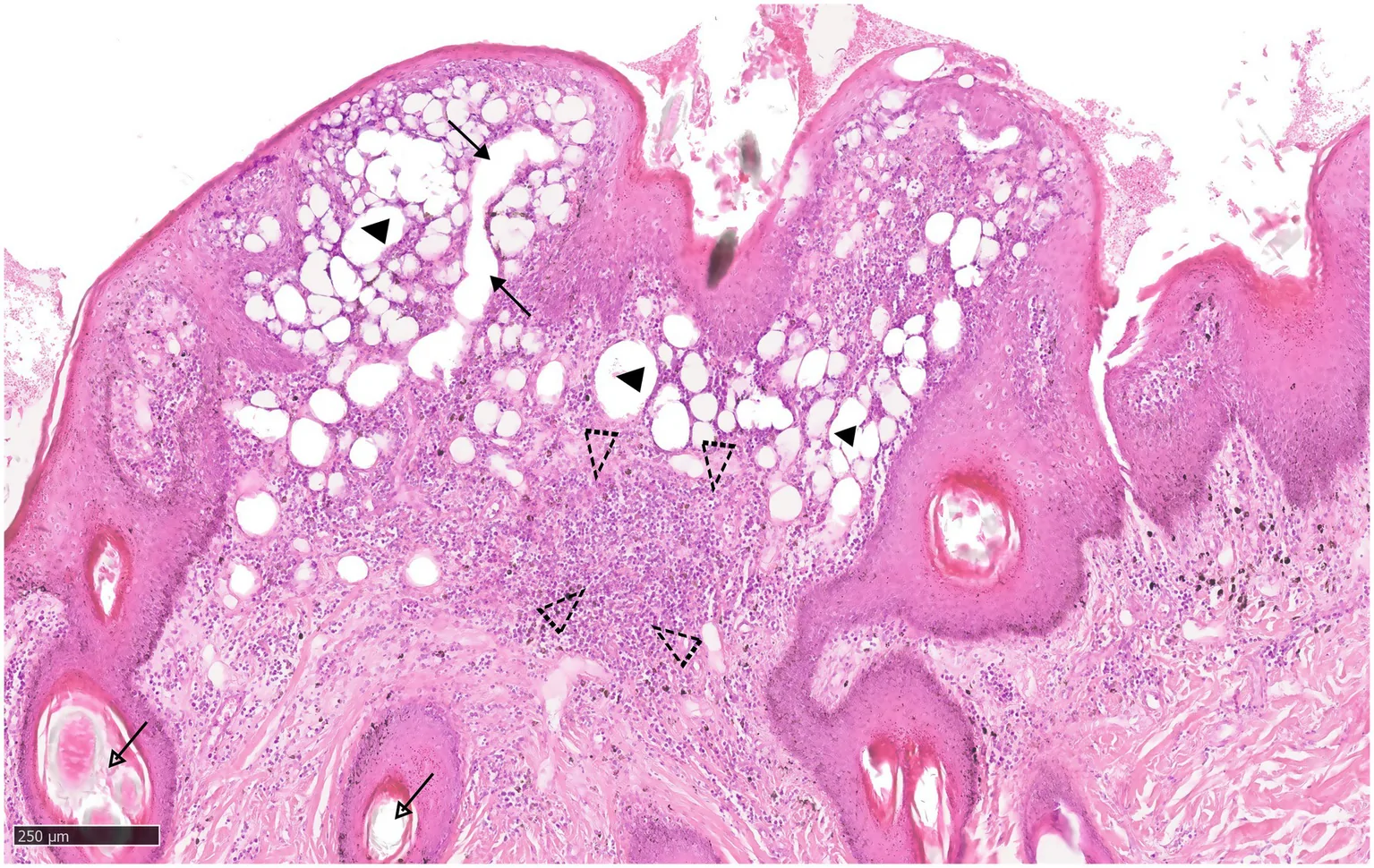
Within the epidermis, there are hair follicles (empty arrow) and multifocal protruding areas showing a proliferation of well-differentiated adipocytes (black arrow head). Multifocally, there is an infiltration of lymphocytes and plasma cells (empty arrow heads) in a vascular stroma (black arrows). (hematoxylin and eosin stain, scale bar: 250 μm).

### 2.1 Histopathology

Histologically, all cases showed similar features (Table 1): these lesions showed a focal, non-encapsulated expansion of the dermis, elevating the overlying epidermis. This was caused by the presence of well-differentiated adipocytes in the dermis arranged in lobules and separated by fibrous septa. Small vessels were scattered throughout the tissue; however, no significant increase in vascularity was observed. The adipocytes typically contained a single clear vacuole located in the center of the lesion, ranging in diameter from 50 to 90 μm, with peripherally displaced nuclei. In the periphery, the vacuoles were occasionally smaller, ranging from 30 to 50 μm in diameter. All lesions were moderately cellular, with no mitotic figures observed in 10 high-power fields (2.37 mm^2^). In case 1, a mild orthokeratotic hyperkeratosis was noted in the overlying epidermis. In case 2, the adipocytes extended down to the level of the hair follicles and grew exophytically. Additionally the adjacent sebaceous glands in the surrounding dermis were mildly hyperplastic.

Cases 3, 4, and 5 showed an exophytic proliferation of portions of the dermal stromal collagen containing numerous well-differentiated adipocytes. In cases 3 and 4, the superficial epithelium lacked adnexa and was focally ulcerated with necrosuppurative inflammation (Figure 1). Additionally, dilated lymphatic vessels were found in case 4. In case 5, no epithelial ulceration was observed.

In contrast to the other four cases, histology of case 6 demonstrated a multinodular appearance (Figure 2). The epithelium was hyperplastic and variably eroded to ulcerated, accompanied by necrosuppurative inflammation with haemorrhagic crusts. Some epithelial cells exhibited ballooning degeneration. In the superficial dermis, numerous lymphocytes and plasma cells were interspersed among mature adipocytes.

In four cases (cases 3–6), no continuity was observed between the dermal adipocytes and those of the subcutis. Conversely, two cases (cases 1, 2) exhibited an indistinct margin separating subcutaneous adipose tissue from the dermal adipocytes.

In cases 1 to 3 and 6, a mild perivascular infiltrate composed of lymphocytes and plasma cells was observed at the periphery. In case 3, occasional eosinophilic granulocytes were additionally identified. Assessment of normal tissue was not possible in cases 4 and 6, due to the limited amount of surrounding unaffected tissue available in these samples.

Special stains for collagen fibers (Goldner and Masson’s trichrome) demonstrated that all samples were permeated by a fine fibrous connective tissue. A mild increase in collagen was found in cases 1 and 5.

## 3 Discussion

In human medicine, nevus lipomatosus cutaneous superficialis (NLCS) is a benign cutaneous hamartoma defined histologically by the presence of clusters of ectopic mature adipocytes within the dermal collagen (2). In veterinary medicine, however, the term “nevus” is controversial. In the WHO International Histological Classification of Tumors of Domestic Animals, the term “hamartoma” is recommended to avoid confusion with pigmented lesions that frequently occur in humans (5). *Hamartoma*, a term that has gained greater acceptance in veterinary pathology, describes a localized accumulation of well-differentiated cells, often arranged irregularly, within a specific region (5). Hamartomas can be further divided into several subtypes: epidermal, follicular, sebaceous, apocrine, and fibroadnexal. The histological features of the lesions in this case series do not correspond to any of the listed subtypes, suggesting that this entity is not represented in the existing classification.

Although the histological and clinical features are largely consistent with those of NLCS, the distribution pattern in the cases presented here differs from the classic presentation in humans. In contrast to the multiple lesions typically observed, solitary-type lesions predominated in the presented cases. Except for a single multinodular case, these lesions were characterized by exophytic growth and significant epidermal protrusion.

In human medicine, the classic form occurs within the first three decades of life, while the solitary form appears between the third and sixth decades of life (6). In the presented cases, these lesions were observed in young to middle-aged animals, including both cats and dogs. Five of the six cases involved canine patients, suggesting a potential species predisposition; however, further investigation with a larger cohort is required to confirm this. Furthermore, the exact age of onset remains unknown, as it could not be determined whether these lesions appeared suddenly or had been present for an extended period.

While a specific 2p24 deletion in one report (7) suggests a congenital origin, the pathogenesis of NLCS remains unclear for the majority of cases, and several other hypotheses have been proposed. Hoffmann and Zurhelle postulated that adipocytes replace degenerated collagen fibers (metaplasia) (2). Furthermore, some authors suspect a perivascular origin; i.e., that the adipocytes derive from pericytes of dermal blood vessels (8). It is also postulated that these lesions arise as a result of abnormal dermal adipogenesis rather than through ectopic fat displacement (9). One study suggests that perivascular mesenchymal progenitor cells possess adipogenic potential and that NLCS originates from these cells (10), while others assume that the cause is idiopathic (11). As the pathogenesis cannot be definitively determined through histological examination alone, further investigations, including immunohistochemical characterization in both canine and feline cases, are necessary to better understand these vascular and cellular alterations.

In humans, predilection sites for the classical (multinodular) form of NLCS are the pelvic girdle, the lumbar region, the buttocks and the upper thigh whereas the solitary form can occur at any cutaneous site (1). In our study, the distribution of the lesions was not restricted to specific anatomical regions but occurred at various skin sites, which is consistent with established findings regarding hamartomas in veterinary medicine and solitary NLCS in human medicine. In humans, a case series from 2026 reported a female predominance in the occurrence of the NLCS (12). In the presented cases, five of the six affected animals were female, while one was male. However, due to the limited number of patients, no conclusion can be drawn regarding a sex-related predisposition. Furthermore, Palekkodan et al. (13) reported a predisposition toward folliculosebaceous hamartomas in large-breed dogs, particularly Labrador Retrievers. Three of the five canine cases presented here involved dogs of medium to large breeds, which is consistent with the occurrence of hamartomas. Treatment is generally not required. Surgical removal may be indicated for cosmetic reasons or if ulceration or other clinical indications are observed (14). While recurrences are rare in humans (4), it is hypothesized that a similar pattern may occur in dogs and cats; however, prospective clinical studies are essential to confirm this assumption and to establish definitive prognostic data.

For a definitive diagnosis, a histopathological examination is necessary. Grossly, the skin lesions should be differentiated from lipoma, fibrolipoma, fibroepithelial polyp, collagenous hamartoma, fibroadnexal hamartoma, sebaceous hamartoma, follicular hamartoma, epidermal hamartoma and sweat gland hamartoma. Presenting primarily as exophytic lesions with marked epidermal protrusion, these cases resemble the solitary form of NLCS, with fibroepithelial polyps as the primary differential diagnosis. According to the World Health Organization International Histological Classification of Tumors of Domestic Animals, these are described as exophytic lesions containing components of both the epidermis and dermis (5). However, as adipocytes are not physiological constituents of the dermis, their presence there is considered ectopic (5); consequently, fibroepithelial polyps do not represent a correct diagnosis for our cases. Because our specimens lacked essential components such as adnexal structures, hair follicles, sweat glands or epidermal cells, fibroadnexal, follicular, sweat gland or epidermal hamartomas must be excluded from the differential diagnosis. While one sample showed sebaceous gland hyperplasia (Case 2)-suggesting a sebaceous gland hamartoma-the WHO International Histological Classification of Tumors of Domestic Animals (5) requires evidence of excessive proliferation, which was not observed in our case. Therefore, these entities can be ruled out.

In human medicine, there is an ongoing debate regarding the distinction between skin tags, NLCS, and lipofibromas. Some authors consider the presence of adipocytes in the dermis of pedunculated lesions to be a defining characteristic of NLCS (15, 16). Others, however, classify these lesions as pedunculated lipofibromas, particularly when they are solitary and occur outside the classic pelvic distribution (17). Histologically, however, the authors saw no differences from NLCS, meaning the distinction was often based on clinical parameters such as patient’s age and location of the lesion (18). The histological presentation in our cases was indistinguishable from NLCS.

The term ‘choristoma’, however, should be avoided, as the subcutis is often regarded as the deepest layer of the skin (19).

Classically, NLCS is defined by a lack of continuity between the dermal adipocytes and the subcutis (2). In two of the cases presented in this study, this distinct boundary was indistinct. While such a loss of continuity might, according to classic dermatopathological criteria, suggest an alternative pathological process or exclude the lesions from a typical NLCS classification, we follow the suggestion of Patil et al. (1). The authors state that the boundary can become increasingly obscured during extensive proliferation of dermal adipocytes, and such cases should nevertheless be considered part of the same entity (1). Due to the indistinct demarcation between subcutaneous fat and dermal proliferation in two of our cases, a lipoma extending into the dermis must also be considered. Further studies are required to establish clear histological boundaries and to better define the distinction between NLCS and dermal lipomas. Even if these two cases were excluded from the analysis, the morphologic characteristics of the remaining cases consistently support our proposed description of the lesion.

Given the overlap of the cases in this study with both NLCS and hamartomatous lesions, the terms ‘superficial lipomatous hamartoma’ or ‘nevus lipomatosus cutaneous superficialis-like lesion’ could potentially serve as descriptive nomenclature for these cases, pending further large-scale studies to validate such a classification.

## 4 Conclusion

This study presents a series of cases of skin lesions exhibiting features consistent with nevus lipomatosus cutaneous superficialis (NLCS) in humans. Considering the evolving understanding of ‘nevus’ and its overlap with hamartomas, our findings suggest the existence of an NLCS-like entity in dogs and cats, which we propose to be provisionally termed ‘superficial lipomatous hamartoma’. Due to the limited number of cases, the findings of this case series should be interpreted with caution; formal validation of this potential entity requires larger-scale studies and integrated clinicopathological correlations.

## Data Availability

The raw data supporting the conclusions of this article will be made available by the authors, without undue reservation.
